# Development of a Real-Time Wearable Humming Detector Device

**DOI:** 10.3390/s24227296

**Published:** 2024-11-15

**Authors:** Amine Mazouzi, Alexandre Campeau-Lecours

**Affiliations:** 1 Department of Mechanical Engineering, Université Laval, Quebec City, QC G1V 0A6, Canada; amine.mazouzi.1@ulaval.ca; 2 Centre for Interdisciplinary Research in Rehabilitation and Social Integration (Cirris), CIUSSSCN, Quebec City, QC G1M 2S8, Canada

**Keywords:** assistive technology, physical disability, speech impairment, humming detection, rehabilitation engineering

## Abstract

This study focuses on the development of a wearable real-time Humming Detector Device (HDD) aimed at enhancing the control of assistive devices through humming. As the need for portable user-friendly tools in assistive technology grows, the HDD offers a non-invasive solution to detect vocal cord vibrations. Vibrations, detected thanks to an accelerometer worn on the neck, are processed in real time using a Fast Fourier Transform (FFT) to identify specific humming frequencies, which are then translated into commands for controlling assistive devices via Bluetooth Low Energy (BLE) transmission. The device was tested with 13 healthy subjects to validate its potential and determine the optimal number of distinct commands that users can achieve. The HDD’s portability and precision make it a promising alternative to traditional voice recognition systems, particularly for individuals with speech impairments.

## 1. Introduction

The rapid advancements in voice recognition technologies have enabled the development of sophisticated home personal assistants, such as Google Assistant and Amazon Alexa. These systems are designed to control smart home devices, including temperature settings, music systems, television displays, and garage doors, through voice commands [1]. While primarily aimed at enhancing convenience in smart homes, voice assistants have also found significant applications in the field of rehabilitation. Indeed, they are increasingly used to control assistive devices like robotic arms and wheelchairs for individuals living with physical disabilities [2].

According to the 2022 Canadian Survey on Disability (CSD), 27% of Canadians aged 15 years and older—amounting to 8.0 million people—reported having one or more disabilities that limited their daily activities [3]. To go further, one in four adults in the United States have some type of disability [4]. However, the effectiveness of voice-controlled assistive technologies heavily relies on the user’s ability to produce clear and comprehensible speech. In fact, many people suffer from speech disorders that hinder their ability to produce complete sentences and, by the same way, effectively use voice-controlled devices. An example of a condition that causes speech problems is neurodegenerative diseases, such as nonfluent aphasia. In Canada, more than 100,000 people live with aphasia, and approximately one third of stroke survivors experience this condition, which significantly reduces speaking speed and grammatical structure [5,6]. In the United States, there are at least 2 million people with aphasia [7]. These are a few examples of people for whom voice assistance is not adapted to their condition.

To address these limitations, alternative methods for controlling assistive devices have been explored. One promising approach is the use of humming sounds instead of speech recognition. Indeed, recent studies have demonstrated the effectiveness of using microphones to detect humming sounds for controlling handheld devices where users can control smartphone applications by intoning different notes through humming [8].

The integration of accelerometers placed against the user’s neck, along with microphones for humming detection, has also been investigated in the context of voice-controlled wheelchairs [9]. The accelerometer detects physical vibrations on the neck that correspond to vocal cord vibrations occurring during speaking or humming. By applying a Fast Fourier Transform (FFT), the fundamental frequency of the signal can be identified and translated into commands for assistive devices. Previous studies have also involved humming detection programs running on computers with external microphones. These programs aimed to enable users to control video games using humming sounds as action commands [10].

Another advantage of using accelerometers is their lower sensitivity to environmental noise, such as the voices of other people, compared to microphones. This reduces interference and simplifies signal analysis, improving the reliability and accuracy of commands. As a result, this approach offers a more accessible and effective alternative to voice control, particularly for individuals with speech impairments.

Despite these advancements, there remains a significant research gap in the understanding of how varying levels of humming intensity can be effectively recognized and utilized for assistive technology. While previous studies have established the feasibility of humming detection, they have largely focused on binary systems (i.e., on/off) or have been limited to stationary setups that lack portability. Furthermore, existing research has not thoroughly examined the realistic capabilities of users to produce multiple notes accurately and consistently, particularly in dynamic environments. The lack of empirical data on the number of distinct notes that individuals with speech impairments can reliably produce leaves a gap in the design and optimization of humming-based assistive devices.

The contributions of this paper are multifaceted. Firstly, it explores humming recognition across different levels of intensity rather than a simple binary on/off system, providing a nuanced approach to signal analysis. Additionally, the proposed system is designed to be portable and operates in real time, offering a practical use of assistive devices. Furthermore, while previous studies demonstrated the possibility of distinguishing different notes, the device was limited to a stationary setup (i.e., a computer), restricting its use to fixed environments. In comparison, the device proposed in this paper uses a microcontroller, which means that the algorithm must operate in real time on a system with limited resources compared to a standard computer. Finally, this study includes a preliminary investigation with participants to determine the realistic number of notes that a participant can effectively manage (i.e., how many notes can the participant accurately produce on command without making a mistake between two notes), addressing a previously unexplored knowledge gap. This comprehensive approach advances the field by integrating detailed analysis, practical usability, and empirical validation.

## 2. Objectives

The goal of this project is to empower individuals living with disabilities to enhance their control of assistive technologies (e.g., robots, wheelchairs) using their voice, ultimately improving their quality of life. The objective is to develop a tool that allows users to control various connected devices, such as room lights or the motor of a wheelchair, through humming. To achieve this, the project aims to design and develop a portable, real-time Humming Detector Device (HDD) that detects humming notes from signals received by an accelerometer.

## 3. Development and Methodology

This section describes the components of the HDD and their assembly, the humming detection algorithm, and the tests that were used to evaluate the efficiency of the HDD with participants.

### 3.1. Components and Assembly System

**Figure 1:**
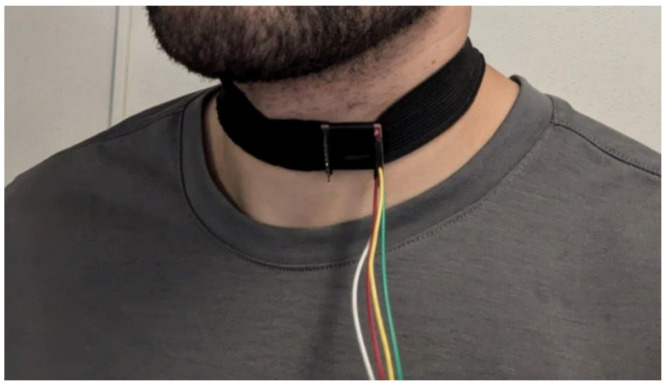
Installation of the accelerometer collar around the neck.

As stated in the previous sections, humming detection will be achieved using an accelerometer placed around the neck. This placement allows the component to detect vocal cord vibrations produced by humming through the skin. To accurately distinguish different humming notes, the accelerometer must have a sample rate high enough to capture these nuances (the humming detection algorithm and the importance of a high acquisition frequency will be explained in Section 3.2). The chosen accelerometer model for this task is the LSM9DS1 9 DOF I2C, which has a configurable sample rate of up to 952 Hz and is compact. The accelerometer is positioned around the neck, just below the Adam’s apple, thanks to a collar, as shown in Figure 1. The collar was designed to eliminate any direct contact between the electronic components and the skin to avoid issues related to patient comfort. We will continue to consider comfort as a priority in future design iterations and user testing.

**Figure 2:**
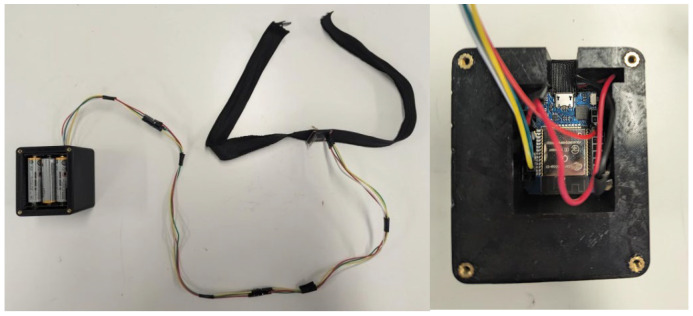
System assembly of the humming detector device.

To collect and process the data, the accelerometer module is connected with long wires to an ESP-WROOM-32 microcontroller, which is powered by a set of three AA batteries. The system assembly is shown in Figure 2.

The AA batteries and the ESP-WROOM-32 are housed in a compact case, which is designed to be conveniently worn on a belt or placed in a pocket or handbag. A cable connects this case to the collar. This setup is used as a proof of concept to validate the feasibility of the device. Indeed, eventually, all the elements (accelerometer, microcontroller, battery) should be miniaturized and integrated into the collar.

### 3.2. Humming Detection Algorithm

**Figure 3:**
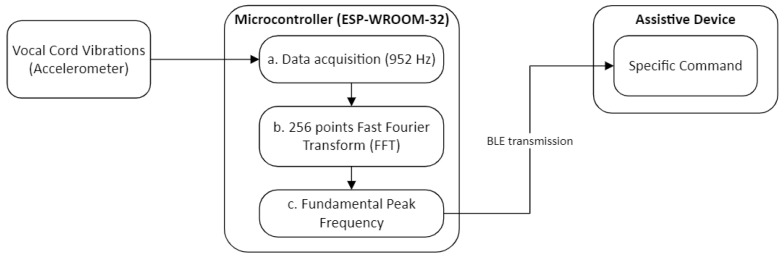
Functional scheme of the Humming Detector Device.

Once the collar is installed on the user, the HDD acquires vocal cord vibration signals. Signal acquisition occurs continuously through the LSM9DS1 accelerometer at a frequency of 952 Hz. The data are collected by the ESP-WROOM-32 microcontroller, which then applies a Fast Fourier Transform (FFT) to every one of the 256 data points acquired by the accelerometer. This process produces the full frequency spectrum of the signal. An algorithm, detailed below, then detects the peak frequency, identifying the fundamental frequency of the signal to be used as a command indicator. This command is then transmitted to an assistive device via Bluetooth Low Energy (BLE). Figure 3 illustrates the functional scheme of the HDD’s overall operation. A more detailed description of each step in the humming recognition process will follow this paragraph.

**Figure 4:**
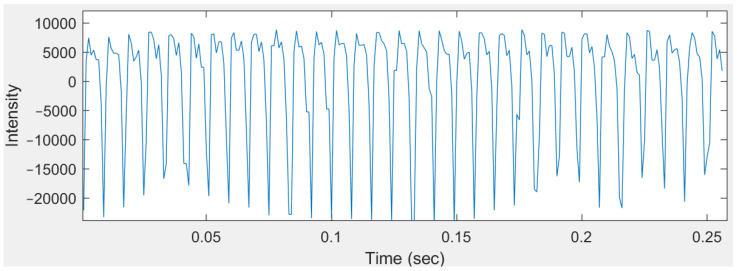
Time signal of the “Do” humming note.

(a) The data acquisition from an accelerometer for a humming note involves capturing the vibrational signals generated by the humming sound over time. When a person hums a note, the accelerometer measures the resulting vibrations into a time-domain signal. This signal typically displays a periodic waveform corresponding to the fundamental frequency of the hummed note and its harmonics. Ideally, the signal appears as a smooth, periodic oscillation with consistent amplitude and frequency. The waveform features a primary oscillation representing the fundamental frequency of the hummed note, with additional smaller oscillations superimposed, representing the harmonics. Figure 4 illustrates an example of the signal acquisition results for a “Do” humming note.

**Figure 5:**
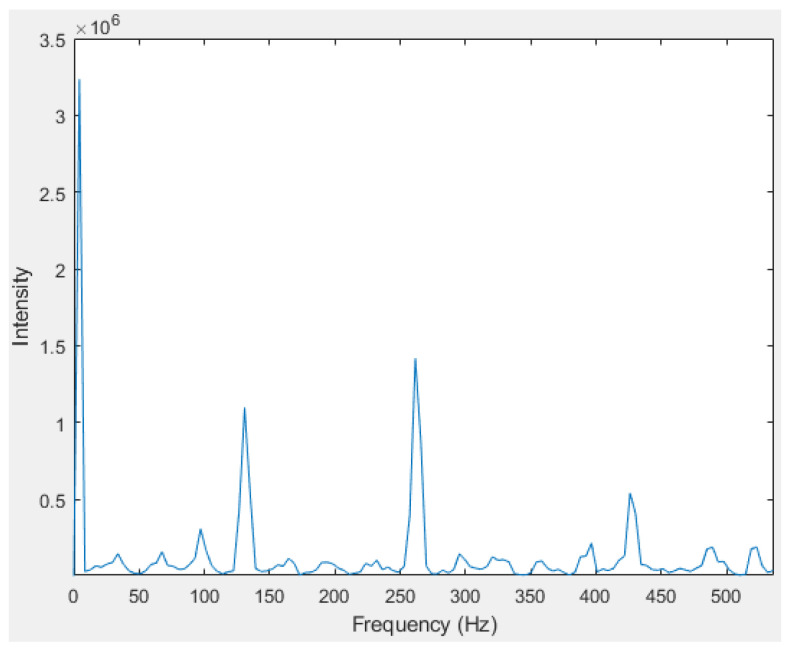
Frequency spectrum of the “Do” humming note.

However, real-world data acquisition is often affected by noise, which can distort the signal. Noise can originate from various sources, such as ambient vibrations, electrical interference, and motion artifacts. In the accelerometer signal, this noise manifests as random fluctuations and irregularities, potentially obscuring the true characteristics of the humming note. Instead of a clean, periodic waveform, the signal may appear jittery or contain unexpected spikes and dips. During the FFT process, noise will typically appear as low-frequency peaks, which can be detected and eliminated, as shown in Figure 5.

(b) For humming detection, the accelerometer data signal is processed using a 256-point Fast Fourier Transform (FFT) to convert the time-domain signal into its frequency spectrum. This process starts by collecting a segment of accelerometer data, which represents vibrations over a short, fixed time period. The FFT decomposes the time-domain signal into its constituent frequencies through mathematical transformations. It computes the amplitude and phase of each frequency component present in the signal. The result is a frequency spectrum that displays the intensity of various frequencies within the signal, with peaks indicating dominant frequencies, such as the fundamental frequency of the ‘Do’ humming note and its harmonics, as shown in Figure 5.

A 256-point FFT generates a frequency spectrum with 256 discrete frequency bins, each representing a specific frequency range. The resolution of these bins is determined by the sample rate of the accelerometer. For example, with a mean sample rate of 1024 Hz, the frequency resolution is 1024/256 = 4 Hz per bin. This resolution allows for the precise identification of the fundamental and harmonic frequencies of the humming note. The frequency spectrum produced by the FFT provides a clear visualization of these peaks, which is crucial for distinguishing humming from other vibrations.

Figure 5 shows the frequency spectrum for a ‘Do’ humming note, revealing three distinct peak frequencies. Typically, a signal corresponding to a ‘Do’ humming note, as well as other notes, exhibits multiple peak frequencies, which include the fundamental frequency and its harmonic frequencies. These harmonics, such as the second and third harmonics, result from the complex vibration patterns of the vocal cords and resonant cavities in the human body. The presence of these multiple peak frequencies is characteristic of musical notes and is crucial for distinguishing different tones and pitches in signal analysis.

Another important consideration for accurate peak frequency recognition is the accelerometer’s sample rate. The sample rate is vital for detecting FFT peak frequencies due to the Nyquist frequency, which is half of the sample rate. According to the Nyquist theorem, to accurately capture and represent a signal without aliasing, the sample rate must be at least twice the highest frequency present in the signal. If the sample rate is too low, frequencies above the Nyquist frequency will be misrepresented, causing aliasing. This misrepresentation can compromise the detection of FFT peak frequencies, leading to an inaccurate identification of both the fundamental and harmonic frequencies. Therefore, to ensure the reliable detection of peak frequencies associated with a humming note, the accelerometer must have a sufficiently high sample rate—ideally at least twice the highest expected harmonic frequency—to avoid aliasing and accurately capture the complete frequency spectrum.

**Figure 6:**
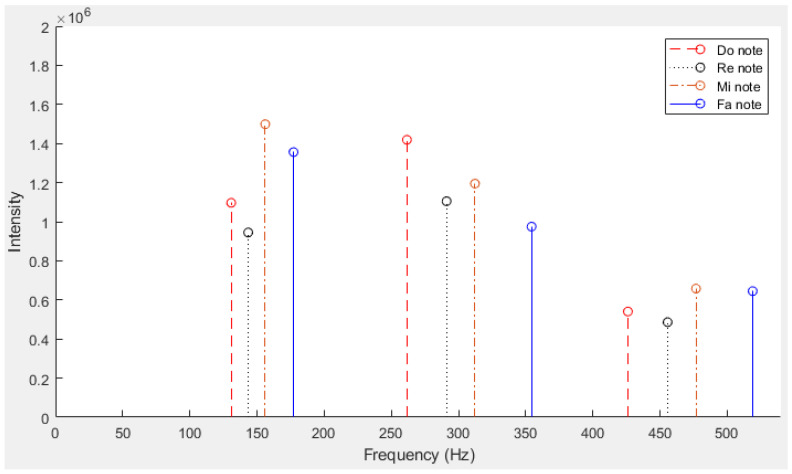
Fundamental and harmonic frequencies of each humming note obtained from trials.

For this study, frequency spectra were generated for each solfège note (“Do”, “Re”, “Mi”, “Fa”, “Sol”, “La”, and “Si”). Multiple tests were performed to confirm that the peak frequencies for each note remained consistent across different trials. Figure 6 illustrates that the peak fundamental and harmonic frequencies increase with higher-pitched notes. Notably, the “Fa” humming note was the highest-pitched note with a third harmonic frequency still below the Nyquist frequency, making it the last note unaffected by sample rate limitations. Table 1 presents the fundamental and harmonic frequency results for the notes “Do”, “Re”, “Mi”, and “Fa” obtained after humming trials performed by a single male person. Obviously, the obtained fundamental frequencies must not be considered as standards for everyone, since it can vary from one person to another depending on each voice’s intonation.

(c) An algorithm for peak frequency detection was developed to identify the fundamental frequency of the emitted humming note. The peak frequency detector algorithm operates as follows for a signal similar to the one in Figure 5:

1. **Compute the Spectrum**: Analyze the frequency spectrum of the signal to identify the amplitude at each frequency.
2. **Find Zero-Crossings in Derivative**: Calculate the derivative of the amplitude as a function of frequency to detect changes in the slope.
3. **Detect Peaks**: Identify points where the derivative changes from positive to negative, indicating potential peaks (maxima) in the frequency spectrum.
4. **Apply a Threshold**: Filter out peaks that fall below a specified amplitude threshold to focus on significant peaks.
5. **Peak selection**: Select the first peak detected and the frequency associated with it.
6. **Fundamental frequency evaluation**: Set an amplitude threshold to half of the amplitude’s value of the peak frequency detected in the previous step. Repeat steps 1 to 5 using data from 0 up to the peak frequency lastly detected. Check if additional peaks are present over another less strict threshold. If no other peaks are found, the detected peak from the first selection will be kept and considered as the fundamental frequency. Otherwise, the first peak detected on the second selection is chosen.

This approach helps in identifying the right fundamental frequency with a low marge of error.

Additionally, to avoid false positives, the detected peak frequency must exceed a predefined intensity threshold. This threshold ensures that only significant signals are processed, filtering out any potential noise or insignificant vibrations that could otherwise lead to erroneous command inputs.

Afterward, the detected peak frequency is categorized to a note (e.g., Do, Re, Mi or Fa). To ensure accurate recognition, the peak frequency must fall within an interval of ±5 Hz of the fundamental frequency for the specific humming note as identified in Table 1.

### 3.3. Translation of Humming Note into Assistive Device Command

**Figure 7:**
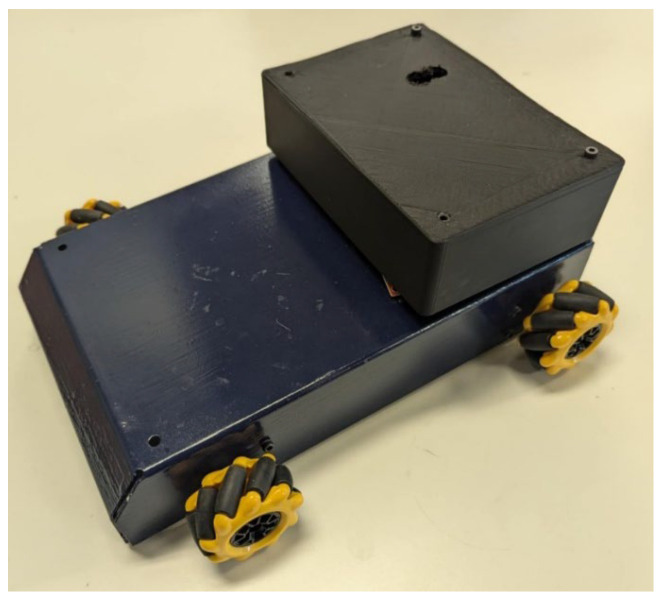
RC car control with humming detector device.

Once a note is detected, the said note category is sent to an assistive device by Bluetooth. In this paper, for testing purposes, the user controls an RC car (see Figure 7). The process involves converting a detected humming note into a specific command for the RC car. Each identified humming note is matched to a predefined set of musical notes, with each note corresponding to a distinct command for the car, as detailed in Table 2. The identified note is then encoded into a digital command and transmitted to the RC car via BLE.

In addition, for false positives due to incidental speech, the system requires the peak frequency associated with a specific note to be detected at least three consecutive times. This introduces a brief delay of less than one second between the humming production and the execution of the command, but it ensures the accuracy and reliability of the commands sent.

Upon reception, the car’s control system decodes and executes the command. This approach demonstrates the potential of using humming as a simple, non-invasive control mechanism for assistive devices, thus enhancing accessibility and user interaction.

### 3.4. LED Lighting Platform Humming Test

**Figure 8:**
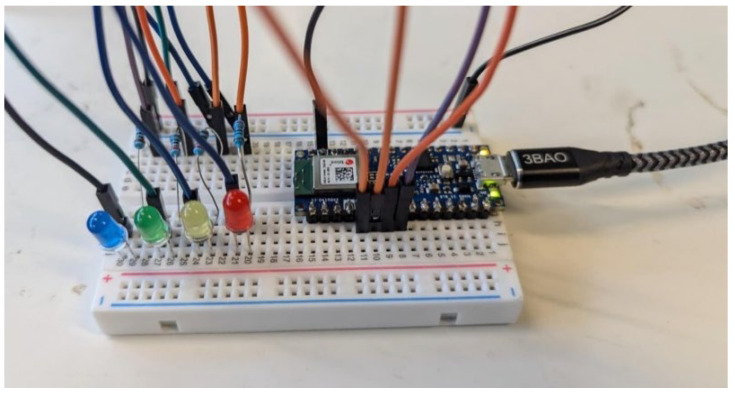
LED montage for humming tests.

To further test the effectiveness of humming recognition with different people, an LED lighting platform (see Figure 8) was developed. The goal here is to know how many distinct humming sounds people can produce reliably on command to know how many distinct commands should be incorporated in the HDD. In the case of this study, for men, the HDD holder will have to produce a humming note that has a fundamental frequency between 100 and 125 Hz for the red LED, 125–150 Hz for the yellow LED, 150–175 Hz for the green LED and 175–200 Hz for the blue LED. For women, it is 200–225 Hz for red light, 225–250 Hz for yellow light, 250–275 Hz for green light and 275–300 Hz for blue light. This change is explained by the fact that women tend to naturally produce a higher pitch voice than men. Each participant had familiarization time to try humming sounds to become familiar with the device. They then tried to light each LED on command at least three times. At the end of the experiment, each participant received a score based on the number of LEDs they were able to activate independently. For example, a participant who successfully activated all four LEDs received a score of 4 out of 4, while a participant who lights up three LEDs received a score of 3.

## 4. Results and Discussion

### 4.1. RC Car Control by Humming Results

The qualitative results from the RC car control experiments using humming notes demonstrated the efficacy and accuracy of the system. The RC car responded reliably to specific humming notes, executing commands with accuracy. Each humming note consistently triggered the intended command, reflecting accurate recognition of the fundamental frequency. Importantly, the system successfully ignored extraneous vocalizations and physical movements, indicating its robust ability to differentiate between relevant humming and irrelevant noise or motion.

The Humming Detector Device also shows a good response time, taking just 0.27 s per iteration to collect data, detect the humming note, and transmit the command via Bluetooth Low Energy (BLE) (see Table 3). This efficiency enables smooth control of assistive devices with quick real-time responsiveness, validating the system’s effectiveness as a reliable and non-invasive control method.

### 4.2. LED Lighting Platform Humming Test Results

**Figure 9:**
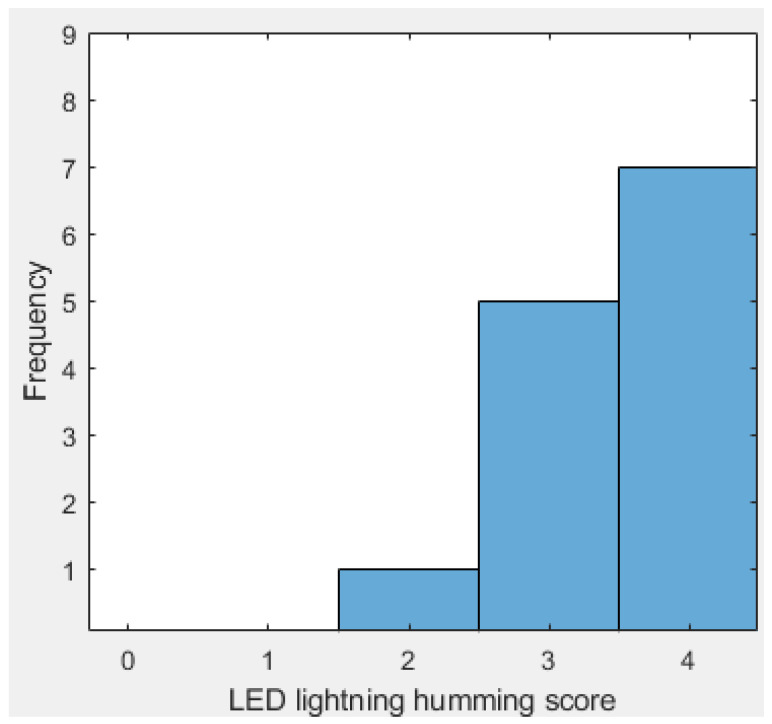
Frequency of each LED lighting platform humming score.

A total of 13 candidates (nine men and four women) participated in the LED lightning humming test. The participants were primarily staff members of the Laval University Robotics Laboratory in good health. Although this sample is not representative of the target population for the HDD, this test allowed our team to verify the device’s efficiency in detecting the correct note. Testing with actual clinical patients will be the next step in the project’s development. This study was approved by the local ethics committee (CIUSSS-CN; project #2019-1603, RIS_2018-616). Each participant received the same instructions as described in Section 3.4. At the end, as it can be seen in Figure 9 and Table 4, 12 out of 13 candidates reached a score of at least 3 out of 4, and 7 candidates (54% of the subjects) reached a perfect score. For the people who did not reach a perfect score, the main issue was to produce a hum from one note to another (e.g., “Do” to “Re”) which results in a deviation of peak frequency that will skip the frequency interval target. For instance, a person who succeeds to light the red light and then tries the next higher pitch hum will unwittingly light the green light.

Overall, this experiment suggests that the system is effective for a diverse range of users. The high success rate also highlights the potential of humming recognition as a viable input method for assistive device control through humming. For now, at least three or four commands can be effectively controlled. From what was observed during the experiments, the authors of this paper believe that with training, the number of notes participants could efficiently manage with precision could increase.

It is important to note that this test was conducted with healthy participants, meaning they do not represent individuals who might use this device for rehabilitation purposes. While the results are promising in terms of accuracy and user interaction, the effectiveness of this system for therapeutic or rehabilitative use remains uncertain. The non-clinical nature of the test subjects might limit the generalizability of these findings to those with physical or cognitive impairments. Therefore, further testing with clinical populations is necessary to fully understand the potential benefits and limitations of this humming recognition system in a rehabilitation context.

## 5. Conclusions

This study demonstrates the feasibility and effectiveness of using humming as a non-invasive input method for controlling assistive devices. The Humming Detector Device (HDD) successfully identified and translated specific humming notes into commands for an RC car and an LED lighting platform, showcasing a promising alternative to traditional voice recognition systems. The results from the LED lighting platform humming test, where 12 out of 13 participants achieved high accuracy (a score of at least 3 out of 4), indicate that the HDD can reliably distinguish between different humming frequencies in real time, allowing users to control devices with precision.

From what was observed during the experiments, the authors of this paper believe that with training, the number of notes participants could efficiently manage with precision could increase. This suggests that the HDD offers many more control options than a simple On–Off command, although it may not be capable of offering a large number of commands (e.g., 10).

In the next iteration of the HDD, our team will aim to expand the number of reachable assistive device commands by either increasing the peak frequency range for a single function (to facilitate the user’s access to a specific function) or introducing a double hum command (e.g., produce a “Do Do” hum to activate a particular feature) similarly to Morse code [11]. Our team is also considering a machine learning approach to enhance speech recognition, aiming for a more versatile and accurate method. This could improve the accuracy rate of the HDD for various patient profiles and enhance the functionality of the assistive devices.

While the study primarily involved participants without clinical profiles, the results are encouraging and suggest that humming recognition technology has significant potential for broader applications, including rehabilitation. The success rate among participants indicates that with further refinement, this system could be adapted to meet the needs of individuals with speech impairments or other disabilities. Future research, especially involving clinical populations, could unlock the full potential of this technology, making it a powerful tool in assistive and rehabilitative care. The next step of the development of this project will be then to repeat the LED lighting platform humming test with the target population during a clinical study. Furthermore, the design of the HDD will be reviewed to increase its portability and its ease of use for the user. Indeed, eventually, all the components (accelerometer, microcontroller, battery) should be miniaturized and integrated into a wearable collar or similar device, which will also be adapted for use with assistive robots and wheelchairs.

## Figures and Tables

**Table 1:** Fundamental and harmonic frequencies of each humming note obtained from trials.

Humming Note	Fundamental Frequency	First Harmonic	Second Harmonic
Do	131 Hz	262 Hz	426 Hz
Re	143 Hz	291 Hz	456 Hz
Mi	156 Hz	312 Hz	477 Hz
Fa	177 Hz	354 Hz	519 Hz

**Table 2:** Humming commands for the RC car.

Humming Note	Associated Command
Do	Car goes forward
Re	Car goes backward
Mi	Car turns left
Fa	Car turns right

**Table 3:** Operation time for each humming recognition steps.

Humming Recognition Step	Time (in s)
Data acquisition	0.240 s
FFT process and fundamental frequency detection	0.008 s
Mean time for BLE data transmission	0.022 s
Total operation mean time	0.270 s

**Table 4:** Results of LED lighting platform humming test.

Mean Score	Median Score	Minimum Score	Perfect Score Proportion	Men: Women Ratio
3.46	4	2	54%	9:4

## Data Availability

The data presented in this study are available on request from the corresponding author. The data are not publicly available due to privacy.

## References

[1] Caranica A., Cucu H., Burileanu C., Portet F., Vacher M. Speech Recognition Results for Voice-Controlled Assistive Applications. Proceedings of the 2017 International Conference on Speech Technology and Human-Computer Dialogue (SpeD).

[2] Pulikottil T.B., Caimmi M., D’Angelo M.G., Biffi E., Pellegrinelli S., Tosatti L.M. A Voice Control System for Assistive Robotic Arms: Preliminary Usability Tests on Patients. Proceedings of the 2018 7th IEEE International Conference on Biomedical Robotics and Biomechatronics (Biorob).

[3] Statistics Canada Disability Rate in Canada Increased in 2022. https://www.statcan.gc.ca/o1/en/plus/5980-disability-rate-canada-increased-2022.

[4] Centers for Disease Control and Prevention Disability Impacts All of Us [Infographic]. Disability and Health Data System (DHDS). https://www.cdc.gov/ncbddd/disabilityandhealth/pdf/disability-impacts-all-of-us-infographic.pdf.

[5] Provincial Health Services Authority Learn More About Aphasia During Aphasia Awareness Month. http://www.phsa.ca/phsa-news-site/Pages/Learn-more-about-aphasia-during-Aphasia-Awareness-Month.aspx#:~:text=More%20than%20100%2C000%20Canadians%20live,of%20stroke%20survivors%20experience%20it.&text=Aphasia%20is%20often%20misunderstood.,it%20does%20not%20affect%20intelligence.

[6] Gunawardena D., Ash S., McMillan C., Avants B., Gee J., Grossman M. (2010). Why Are Patients with Progressive Nonfluent Aphasia Nonfluent?. Neurology.

[7] National Aphasia Association Aphasia Statistics. https://aphasia.org/aphasia-resources/aphasia-statistics/#:~:text=INCIDENCE%20OF%20APHASIA&text=About%205%2C000%2C000%20people%20survived%20strokes,in%20the%20USA%20with%20aphasia.

[8] Sook Young W., Lee D.I., Smith J. (2007). Humming Control Interface for Hand-Held Devices. Proceedings of the 9th International ACM SIGACCESS Conference on Computers and Accessibility (Assets ‘07).

[9] Peixoto N., Nik H.G., Charkhkar H. (2013). Voice Controlled Wheelchairs: Fine Control by Humming. Comput. Methods Programs Biomed..

[10] Hainisch R., Platz M. Phonetic Control: A New Approach for Continuous, Non-Invasive Device Control Using the Vocal Tract. Proceedings of the 2007 IEEE 10th International Conference on Rehabilitation Robotics.

[11] Schweitzer F., Campeau-Lecours A. (2021). IMU-Based Hand Gesture Interface Implementing a Sequence-Matching Algorithm for the Control of Assistive Technologies. Signals.

